# Early onset obesity and adrenal insufficiency associated with a homozygous POMC mutation

**DOI:** 10.1186/1687-9856-2011-5

**Published:** 2011-07-06

**Authors:** Meenal S Mendiratta, Yaping Yang, Andrea E Balazs, Alecia S Willis, Christine M Eng, Lefkothea P Karaviti, Lorraine Potocki

**Affiliations:** 1 Endocrinology section-Department of Pediatrics, Baylor College of Medicine, One Baylor Plaza, Houston, TX 77030, USA; 2 Department of Molecular and Human Genetics, Baylor College of Medicine, One Baylor Plaza, Houston, TX 77030, USA; 3 Texas Children's Hospital, Houston, TX 77030, USA

## Abstract

Isolated hypocortisolism due to ACTH deficiency is a rare condition that can be caused by homozygous or compound heterozygous mutations in the gene encoding proopiomelanocortin (*POMC*). Loss of function mutations of *POMC *gene typically results in adrenal insufficiency, obesity and red hair. We describe an 18 month old Hispanic female with congenital adrenal insufficiency, a novel *POMC *mutation and atypical clinical features. The patient presented at the age of 9 months with hypoglycemia and the endocrine evaluation resulted in a diagnosis of ACTH deficiency. She developed extreme weight gain prompting sequence analysis of *POMC*, which revealed a homozygous c.231C > A change which is predicted to result in a premature termination codon. The case we report had obesity, hypocortisolism but lacked red hair which is typical for subjects with *POMC *mutations. Mutations of *POMC *should be considered in individuals with severe early onset obesity and adrenal insufficiency even when they lack the typical pigmentary phenotype.

## Introduction

Congenital isolated ACTH deficiency is a rare condition. It is characterized by decreased plasma adrenocorticotropic hormone (ACTH) concentration resulting in adrenal insufficiency and consequently low serum cortisol concentration and lack of hyperpigmentation. These subjects show no ACTH response to exogenous corticotrophin releasing hormone (CRH) administration and have a normal pituitary morphology. Additionally, the other pituitary hormones are normal, unlike secondary adrenal insufficiency which can be associated with multiple pituitary hormone deficiencies. Congenital isolated ACTH deficiency may present in early neonatal period or in later childhood.

ACTH is produced from the precursor molecule proopiomelanocortin (POMC), by the action of the cleavage hormone pro-hormone convertase 1 (PCSK1) when stimulated by CRH. The genes associated with congenital isolated ACTH deficiency are *TBX19 *(*TPIT*), *PCSK1 *(*PC1*) and *POMC*. Genetic mutations in CRH or its receptor have not been associated with isolated ACTH deficiency [1]. *TBX19 *is a cell restricted transcription factor required for terminal differentiation of pituitary corticotrophs and for expression of POMC. Typically, individuals with *TBX19 *mutations present with neonatal hypoglycemia and cholestatic jaundice and account for 73% of isolated ATCH deficiency [2]. *PCSK1 *(*PC1*) mutations are rare and have been described in two cases with obesity, abnormal glucose homeostasis, ACTH and gonadotropin deficiency and gastrointestinal abnormalities [3]. The classical clinical triad described with POMC mutations includes early onset obesity, hypocortisolism and red hair.

We describe an individual with early onset obesity, adrenal insufficiency and a novel POMC mutation but without the typical pigmentary phenotype of red hair and pale skin.

## Case Report

A 9 month old female presented with hypoglycemia and hyponatremia following a three day history of fever, emesis, poor oral intake and progressive lethargy. She was the first born child of nonconsanguinous parents of Hispanic ethnicity whose family history was noncontributory. Both parents were of average build (father's BMI 25.8 and mother's 24.5). Mother received routine prenatal care and there were no complications during the pregnancy. She was born appropriate for gestational age at 38 weeks via spontaneous vaginal delivery and was in the hospital for a week for apnea and neonatal jaundice. Her past history was also significant for delayed motor milestones. She could not sit without support, nor did she crawl or roll over but had a pincer grasp.

On the day of presentation, she became increasingly lethargic and was taken to a hospital where she was found to be hypoglycemic (blood glucose 1.2 mmol/L) and hyponatremic (Na 121 mmol/L). Her weight was 11.5 kg, head circumference 47 cm (both above the 95th percentile) while her length was 72 cm (at the 75^th ^percentile). Craniofacial features were notable for dark hair and dark roots, broad forehead with mild frontal bossing. Head and neck examination revealed a right sided otitis media and fundoscopy was normal with no signs of optic atrophy. Skin examination showed no hyperpigmentation of nipples or palmer creases. The remainder of her physical examination was normal. She was stabilized on intravenous fluids. Both her blood glucose and sodium levels normalized. Urinalysis and culture were diagnostic of a urinary tract infection. Two days after admission, she developed a right sided pleural effusion and moderate ascites. As her condition worsened, she required intubation and intensive care. A random cortisol level was low prompting a 1 mcg ACTH stimulation test which revealed no response with a low ACTH level. Thyroid function studies and growth factors were normal (Table 1). MRI of the brain revealed a normal pituitary morphology.

**Table 1:** Laboratory evaluation

Analyte	Patient Value	Normal range for age
Random Cortisol nmol/L	19.31	77.25-634

Stimulated Cortisol nmol/L	27.29	> 496.6

ACTH pmol/L	< 1.1	1.1-5.94

TSH mU/L	3.6	0.32-5

T4 nmol/L	97.81	89.6-193

IGF-1 mcg/L	46.l	25- 265

IGF-BP3 mg/L	1.8	0.7-3.6

Secondary adrenal insufficiency was clinically suspected due to the lack of hyper-pigmentation. As primary adrenal insufficiency was not under consideration, renin and aldosterone levels were not obtained. Hyponatremia may be seen in severe cortisol deficiency due to decreased free water clearance. Congenital isolated ACTH deficiency was diagnosed based on her clinical presentation, absent hyperpigmentation, low basal and stimulated cortisol levels and normal levels of other pituitary hormones. Exon sequencing of *TBX19 *was normal. She recovered with stress dose steroids and intravenous antibiotics and was discharged on oral maintenance glucocorticoid replacement with hydrocortisone (10 mg/m^2^/day) and blood glucose monitoring.

**Figure 1:**
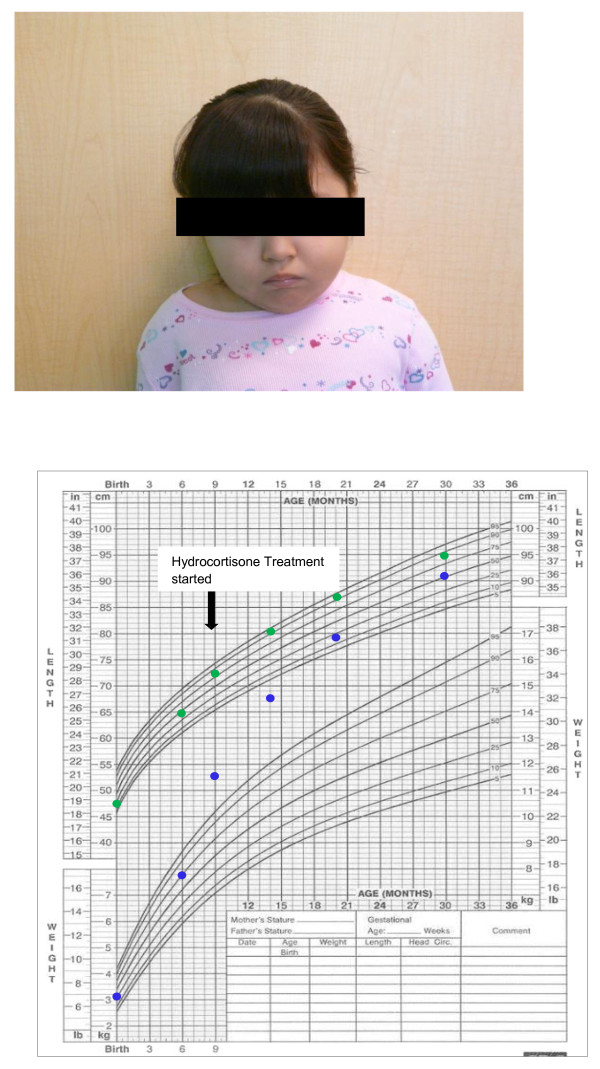
**Patient (age 30 months) and her growth chart**.

**Figure 2:**
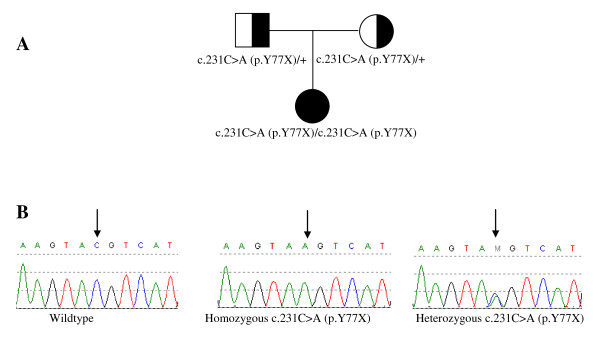
**Patient pedigree and *POMC *gene mutation analysis**. (A) Pedigree of the nuclear family with mutation status noted. (B) Sequence chromatograms: wildtype sequence (left) with a C at nucleotide position 231 of the POMC gene, patient (middle) with homozygous substitution of A for C resulting in tyrosine at amino acid position 77 (p.Y77x), a premature stop codon and parental sequence (right) demonstrates heterozygote C to A substitution represented by the letter M (arrow).

During follow up, the mother was concerned about the child's increased appetite and weight gain (Figure 1) and reported normal blood glucose levels at home. Although the patient did not have red hair, gene sequencing of *POMC *was performed due to her obesity and adrenal insufficiency. *POMC *gene sequencing showed a homozygous c.231C > A change which causes a premature termination codon. Each parent was heterozygous for this mutation (Figure 2).

## Discussion

Individuals with adrenal insufficiency usually present with weight loss, fatigue, hypoglycemia, severe infections and shock. Our subject was diagnosed with adrenal insufficiency due to isolated ACTH deficiency, based on her clinical presentation and endocrinology evaluation. She was obese on initial examination and continued to gain weight. Iatrogenic hypercortisolism as a cause of her abnormal weight gain was not suspected as her linear growth did not deviate from her established percentile curve (Figure 1) and she received normal maintenance steroid dose. The diagnosis of POMC deficiency was confirmed by exon sequencing of the *POMC *gene which showed a novel homozygous mutation. This mutation caused a substitution of cytosine by adenosine at codon 231 resulting in a premature stop codon instead of tyrosine at amino acid position 77 (p.Y77X).

**Figure 3:**
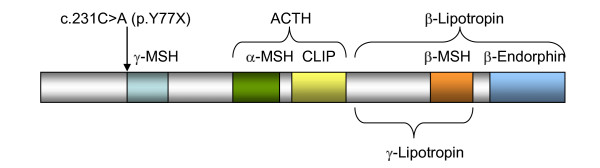
**Proopiomelanocortin protein**. The POMC gene encodes a hormone precursor for distinct melanotropins (MSH), lipotropins, and endorphins including -MSH (amino acids 77-87, shown in light blue), -MSH (amino acids 138-150, shown in green), Corticotropin-like intermediary peptide (CLIP) (amino acids 156-176, shown in yellow), ACTH (amino acids 138-176, in brackets), -MSH (amino acids 217-234, shown in orange), -endorphin (amino acids 237-267, shown in blue), -Lipotropin (amino acids 179-267, in brackets), and Lipotropin (amino acids 179-234, in brackets). The amino acid positions are not drawn to scale. The c.231C>A mutation causes a premature stop within the protein just upstream of the MSH motif.

POMC is a complex pro-peptide, encoded on chromosome 2p23.3. It is expressed in several tissues including the hypothalamus, pituitary gland, skin and the immune system [4]. POMC is processed post-translationally to produce the bioactive peptides: ACTH, β-endorphin, and α-, β-, γ- melanocyte stimulating hormones (MSH) (Figure 3). The action of these melanocortins is mediated through the action of five melanocortin receptors: MC1R to MC5R. MC1R, MC2R and MC5R have been implicated in pigmentation of the skin, adrenal steroidogenesis and thermoregulation respectively [5]. MC3R and MC4R are expressed in the lateral hypothalamic, hypothalamic parvicellular and perifornical areas and play an important role in food intake and energy expenditure [3]. The action of ACTH is well recognized and it is the only known ligand of MC-2R. α-MSH binds to MC1R in melanocytes to stimulate the synthesis of eumelanin (black/brown pigment) [6] and is involved in the control of food intake and energy expenditure through MC3 and MC4 receptors [7]. There is evidence that β- MSH also plays a role in the control of human energy homeostasis [8]. γ-MSH has been implicated in the central control of the cardiovascular system while β-endorphin plays a role in pain perception and analgesia [9].

The first two cases of POMC deficiency were described by Krude et al., in 1998 [10]. These two subjects of Caucasian descent presented with hypoglycemia and neonatal jaundice. They were later diagnosed with ATCH deficiency which was associated with early onset obesity and red hair [10]. Three more individuals described by the same group in 2003 had a similar clinical phenotype [11]. However, Farooqi in 2006 described a two year male of Turkish ethnicity with central hypocortisolism, severe obesity at 6 months and dark colored hair [12]. On closer inspection, they noted that he had brown hair with dark red roots. An 18 year old female of North African ancestry with ACTH deficiency at birth, severe early onset obesity and dark hair was described by Clément in 2008 [13]. At the age of 13 years, she had delayed puberty and on evaluation was found to have hypothyroidism, hypogonadism and growth hormone deficiency (Table 2).

**Table 2:** Previously reported cases of POMC deficiency

Age/Gender	Country/region of origin	Triad criteria (red hair, AI, obesity)	POMC gene mutation	Reference
Neonatal/M	Slovenia	+	A6851T/6996del	Krude[11]

4 weeks/M	Netherlands	+	C3804A/C3804A	Krude[11]

6 months/F	Switzerland	+	C3804A/7100insGG	Krude[11]

2 years/M	Turkey	-	C6906del/C6906del	Farooqi[12]

3 years/F	Germany	+	G7013T/C7133del	Krude[10]

5 years/M	Germany	+	C3804A/C3804A	Krude[10]

18 years/F	North Africa	-	6922insC/6922insC	Clément[13]

Unknown	Japan	unknown	GTG3895AC	Krude[9]

* AI = adrenal insufficiency

POMC knockout (*POMC*^-/-^) mice models show a similar phenotype with adrenal insufficiency, marked obesity and altered pigmentation. These *POMC*^-/- ^mice also have higher fat mass than age-matched normal wildtype (WT) mice and a reduced basal metabolic rate. Their total plasma T4 levels were significantly lower than those of WT mice. Thus, the obesity phenotype may be due to increased food intake and reduced metabolic rate [3]. Administration of α-MSH ameliorated the obesity seen in these mice [7]. Coll's group also demonstrated that *POMC *heterozygous mice (*POMC*^+/-^) had a weight similar to WT mice on standard chow but became obese on high fat chow (45% fat) due to increased food intake [3]. In humans, genetic linkage studies have identified chromosome 2p22 (region encompassing the *POMC *gene) as the site of gene or genes influencing common obesity [14]. Heterozygous mutations in *MC4R *are the commonest known cause of monogenic obesity [3]. Farooqi's group studied the other family members of the Turkish child and found that 11 of the 12 who were heterozygote *POMC *carriers were obese as compared to the one of 7 wild type family members [12]. This strongly suggests that the loss of one copy (haploinsufficiency) of the *POMC *gene predisposes to obesity [6]. Various point mutations of the *POMC *gene have also been shown to increase the risk of obesity in carriers [8,15].

The management of obesity in POMC deficiency remains a challenge. Krude's group administered intra-nasal ACTH _4-10 _to the first two probands in a 3 month trial period [11]. The peptide fragment ACTH _4-10 _is identical to α-MSH and was shown to be effective in reducing body weight in normal weight individuals [16]. However, even with the maximum dose of 5 mg/day, there was no effect on weight, body composition or metabolic rate. This lack of activity was explained by the lower affinity of ACTH _4-10 _to MC4R. These two subjects also had repeated elevation of TSH, borderline reduction of total T4 and normal T3 levels. Both were treated with levothyroxine for a year to normalize T4 and TSH, but again this had no impact on their weight [11].

Melanocortin peptides stimulate both melanogenesis and the switch from pheo (red-yellow) to eumelanogenic (black) pathways [17]. The 18 year old North African subject underwent hair pigment analysis which showed a significant increase in the levels of both eumelanin and pheomelanin. However, there was a reduced ratio of eumelanin to pheomelanin [13]. In mice, POMC inactivation causes a lighter coat on some genetic backgrounds. However, Slominski and his group showed that non-agouti 129;B6 mice with *POMC *deletion, showed normal eumelanin production with no effect on hair color. They speculated that this may be due to ligand independent activity of MC1R which allows synthesis of eumelanin [17]. Similarly, in Caucasians, eumelanin synthesis is more dependent on the presence of POMC derived peptides while in non-Caucasian ethnic groups, other genetic variants may act to maintain eumelanin synthesis [18].

The individual we present is an 18 month old female of Hispanic ethnicity, with POMC deficiency associated with hypocortisolism and early onset obesity, but without the typical phenotype of red hair. She has dark brown-black hair and roots. On closer questioning, the mother stated that her daughter's hair and hair roots have remained unchanged since birth but noted that it has a reddish glint in sunlight. However, we were not able to appreciate this in the fluorescent lighting of the examination room. She has a novel *POMC *gene mutation which may allow expression of hair color or as discussed above, hair pigmentation in non-Caucasians may not be entirely dependent on melanocortin peptides. Since POMC derived peptides have a wide array of biological functions, it is possible that she may develop other manifestations later on in life. In conclusion, *POMC *gene mutations should be considered in patients with early onset severe obesity, adrenal insufficiency even in the absence of red hair, especially in non-Caucasians.

## Consent

Written informed consent was obtained from the patient for publication of this case report and the accompanying images. A copy of the written consent has been included for review by the Editor-in-Chief of this journal.

## Competing interests

The authors declare that they have no competing interests.

## Authors' contributions

MM, LK, AB and LP participated in the care of the patient and drafted the manuscript. YY, AW, CE performed the molecular genetic studies. YY was also responsible for Figures 2 and 3. All authors read and approved the final manuscript.
